# Syllabi collection on diversity and intersectionality in public health: reflecting on the development

**DOI:** 10.1093/eurpub/ckac131.382

**Published:** 2022-10-25

**Authors:** L Wandschneider, D Podar, L Wetzel, H Luetke Lanfer, O Skrypnikova, O Razum, S Selig, Y Namer

**Affiliations:** 1 School of Public Health, Bielefeld University, Bielefeld, Germany; 2 Health Sciences, University of Applied Sciences Fulda, Fulda, Germany; 3 School of Public Health, National University of Kyiv-Mohyla Academy, Kyiv, Ukraine; 4 Department of Public Health and Health Sciences, University of Michigan-Flint, Michigan-Flint, USA

## Abstract

**Background:**

Highlighting the intersectionalities between different markers of diversity and health inequities encourages the reconsideration of normativities in public health (PH). We developed an open access collection of syllabi on the relevance of intersectionality and diversity in PH together with the Association of Schools of Public Health in the European Region (ASPHER).

**Objectives:**

We developed the syllabi in a participatory, iterative process guided by transformative teaching pedagogy. We reflect on this process and how this can inform the enhancement of the syllabi themselves, as well as future curriculum development.

**Results:**

We recruited a core group of 9 PH researchers, teachers and professionals from all career levels from participants of introductory session presentations in different settings (e.g., 14th EPHC, ASPHER Retreat). The core group met once a month for one year online, and each meeting took the form of co-working sessions in breakout rooms to develop the syllabi based on interest and expertise. We designed a qualitative online survey to evaluate and ensure the scientific rigor and pedagogical value of the syllabi. We invited critical and constructive input from ASPHER member school professionals with expertise in intersectionality, diversity or curriculum development in PH in terms of content and pedagogy.

**Conclusions:**

Drawing from the expertise of the PH community we combined diverse professional and cultural backgrounds, experiences from different career levels and PH education systems, as well as specialisation in the PH field. The transformative pedagogical approach was considered particularly valuable in strengthening competences such as reflexive strategies and self-, social- and global awareness which are key to teaching on diversity and intersectionality issues. The peer-review structure supports the uptake in PH education and a sustainable implementation. The collection will also allow PH faculty to diversify their pedagogical approaches.

**Key messages:**

• Inclusion of health inequities, diversity and social injustice issues is crucial in public health curricula, since an intersectional perspective is increasingly acknowledged in public health research.

• The syllabi collection will equip public health teachers of all career levels to develop their own course material on social identities and their significance for public health.

